# Early detection of contrast-induced encephalopathy after mechanical thrombectomy using flat-detector CT: incidence, risk factors, and clinical implications

**DOI:** 10.1007/s00234-025-03630-0

**Published:** 2025-05-01

**Authors:** Mousa Zidan, Shiwa Ghaei, Felix Bode, Alexander Radbruch, Franziska Dorn

**Affiliations:** 1 Department of Neuroradiology, University Hospital Bonn, Bonn, Germany; 2 Department of Neuroradiology, LMU-Klinikum der Universität München Medizinische Klinik und Poliklinik IV, Munich, Germany

**Keywords:** Contrast-induced encephalopathy, FDCT, Mechanical thrombectomy, Neurotoxicity

## Abstract

**Purpose:**

Contrast-induced encephalopathy (CIE) is a rare but increasingly recognized complication following therapeutic and diagnostic endovascular neurointerventions, including mechanical thrombectomy (MT). This study aimed to investigate the incidence, imaging characteristics, and risk factors of CIE, utilizing flat-detector (FD) CT for immediate post-interventional assessment.

**Methods:**

We retrospectively evaluated patients who underwent MT for acute ischemic stroke (AIS) between January 2020 and February 2023, who received FDCT directly after the intervention. CIE was diagnosed based on clinical criteria and radiological findings, confirmed with follow-up dual-energy (DE)-CT. Risk factors for CIE were analyzed using logistic regression, and associations with clinical outcomes (discharge mRS and mortality) were assessed.

**Results:**

CIE was identified in 16 patients of 339 (4.7%). Patients who developed CIE required a significantly higher number of device passes (median: 3 vs. 2, *p* = 0.033) and contrast volume exposure (200 mL vs. 110 mL, *p* = 0.017). Cervical ICA occlusion (*p* = 0.025) and intracranial stent angioplasty (*p* = 0.047) were frequent in the CIE group. Logistic regression confirmed the number of device passes as an independent predictor of CIE (OR: 1.51; 95% CI: 1.13–2.01; *p* = 0.005). No significant associations were found between CIE and unfavorable clinical outcomes (mRS > 3, *p* = 0.9) or mortality (*p* = 0.89).

**Conclusion:**

FDCT allows for early detection of CIE-related radiological changes immediately after MT. Procedural complexity, including device passes, was identified as a risk factor. Identifying other risk factors require further investigations, due to the low incidence rate of detected CIE. These findings highlight the need for intensified monitoring for high-risk patients to mitigate the risk of CIE.

## Introduction

Contrast-induced encephalopathy (CIE) is a rare and poorly understood entity which is being increasingly recognized after therapeutic and diagnostic endovascular neurointerventions [1] e.g. coil embolization [2] and deployment of flow diverters [3]. It has been earlier reported in patients undergoing coronary angiography [4]. It has been sparsely documented in the literature with an incidence rate of varying from 0.66% in coil embolization [5] to 1.7% after mechanical thrombectomy (MT) [6]. The number of MTs performed for stroke treatment has been steadily increasing in recent years [7]. Consequently, it is reasonable to anticipate a corresponding rise in cases of post-procedural CIE.

CIE is characterized by the acute onset of neurological deficits. Wide range of clinical manifestations have been described, visual disturbances in form of cortical blindness, seizures, episodes of encephalopathy as well as aphasia and motor or sensory deficits. Both immediate onset following contrast exposure and delayed onset have been reported, with symptoms typically resolving within a few days [6, 8, 9].

CIE is often a diagnosis of exclusion after more pressing causes, such as hemorrhagic events or recurrent ischemia are ruled out, which require prompt therapeutic consequences.

The current literature lacks sufficient evidence to establish formal imaging criteria for diagnosing CIE. However, the most reported abnormal findings in CT are ipsilateral brain edema, cerebral sulci effacement, increased attenuation of cerebrospinal fluid (CSF) as well as cortical enhancement [6, 10].

Currently, there are no established treatment guidelines for CIE. Published case reports indicate a preference for conservative medical management, consisting of corticosteroids and anti-seizure therapy [10].

In many neurointerventional centers, it is considered good clinical practice to perform a post-procedural flat-detector CT (FDCT) to identify potential immediate complications. FDCT has the advantage of detecting findings that may not be apparent on conventional digital subtraction angiography (DSA).

Given the unclear temporal relationship between the administration of iodinated contrast, the onset of neurological deterioration, and abnormal imaging findings, this study aimed to assess the utility of FDCT in detecting imaging findings indicative of CIE immediately after MT in the angiography suite. The findings were correlated with clinically reported neurological worsening and follow-up dual-energy (DE) CT. Furthermore, the study aimed to identify associated risk factors and predictive markers.

## Materials and methods

### Participants and inclusion criteria

We performed a retrospective evaluation of all consecutive patients who underwent MT due to a vessel occlusion and who received post-interventional FDCT between January 2020 and February 2023 in a tertiary stroke center in Germany. Inclusion criteria were (a) patients received post-interventional FDCT. Exclusion criteria were (a) intracranial hemorrhage at baseline imaging; (b) recurrent ischemic stroke within few days; (c) no available follow-up imaging; (d) lack of clinical documentation in the medical records.

### Endovascular recanalizing procedures

MT was performed under general anesthesia using a transfemoral approach, with materials selected based on the treating physician’s judgment and expertise. There were no restrictions regarding the use of stent retrievers or aspiration techniques. The first-line approach at this tertiary center was a combination of stent retriever and distal aspiration thrombectomy. For patients with tandem occlusions, a retrograde approach (addressing the intracranial occlusion first) was preferred, with an antegrade approach (treating the proximal occlusion first) used only if crossing the proximal occlusion proved impossible. Acute carotid artery stenting was performed when indicated.

For patients requiring extracranial stenting, intravenous acetylsalicylic acid (500 mg) was given before stent implantation, followed by dual antiplatelet therapy initiated no earlier than 24 h post-intervention and only after follow-up imaging (CT or MRI).

Eligibility for intravenous tissue plasminogen activator (IV tPA) was assessed, and tPA treatment was administered in accordance with established guideline recommendations.

#### Contrast agent

For the MT, the contrast medium Iomeprol with 300 mg iodine/ml (Imeron 300^®^, Bracco Imaging Deutschland GmbH, Konstanz, Germany) was utilized. Iomeprol is a non-ionic, iodinated, low-osmolar contrast agent with an osmolality of 618 mosmol/kg.

### Image acquisition

#### FDCT

Post-interventional images were acquired in the angio-suite (Allura Xper FD 20 flat-detector with Xper-CT, Philips, Best, The Netherlands). The acquisition parameters have been reported in detail elsewhere [11].

#### Follow-up imaging

In the current investigstion all follow-up scans are obtained with an IQon spectral scanner (Philips Healthcare, Best, The Netherlands). Acquisition parameters have been reported in detail elsewhere [12]. Spectral postprocessing was performed using dedicated software (IntelliSpace Portal, Philips Healthcare, The Netherlands). A virtual non-contrast (VNC) scan, iodine-removed map, and an iodine map were generated. Initial DECT was routinely performed 24 h after MT as part of institutional protocol. However, in cases of clinical deterioration, DECT was conducted earlier to promptly assess for complications. For patients with imaging findings consistent with CIE, a follow-up DECT was typically performed 48–72 h after the initial scan to evaluate the reversibility of changes.

### Diagnosis of CIE: clinical and radiological criteria

CIE is diagnosed when both clinical and radiological criteria are met as follows:

#### Clinical criteria

Previously defined by Chu et al. [6].

1. **Neurological Deterioration**: Defined as an increase of ≥ 4 points in the National Institutes of Health Stroke Scale (NIHSS) or a decrease of ≥ 2 points in the Glasgow Coma Scale (GCS) score.
2. **Delayed Recovery**: A lack of expected neurological improvement following MT, which cannot be explained by the original ischemic, recurrent stroke or hemorrhagic transformation.

#### Radiological criteria

On FDCT performed in the angiography suite, the following changes were identified:

Swelling beyond the Infarct Core extending beyond the primary ischemic area, cerebral sulci effacement and increased attenuation of CSF.

Accompanied with or without:

1. Cortical enhancement.
2. Contrast Staining: defined as hyperdense lesions within the brain parenchyma or subarachnoid space.

To further classify these findings, follow-up DECT was utilized, incorporating VNC scans and iodine-only images:

- **Hemorrhage**: Diagnosed when hyperdense lesions correlate with findings on VNC images.
- **Contrast Exudation**: Identified when hyperdense lesions appear only on iodine-only images, without a corresponding hyperdensity on VNC scans.

### Verification of radiological features associated with CIE

To identify radiological features specific to CIE:

- The observed changes had to be **reversible** as confirmed on additional follow-up DECT.
- The reversibility was accompanied by clinical relief of unexplained neurological deterioration.
- Cases presenting with isolated cortical enhancement alone did not meet the diagnostic criteria for CIE, as these findings were later confirmed on follow-up DECT to represent ischemic-related enhancement.

Patients were excluded if their hyperdense lesions were attributable to:

- Parenchymal enhancement caused by infarction.

### Statistical analysis

We collected data on various demographic characteristics, vascular risk factors, and neurological status. Specifically, we recorded the NIHSS score, as well as the mRS score at admission and discharge. Laboratory variables included blood glucose levels and glomerular filtration rate (GFR). Additionally, we considered patients’ medication history prior to admission, focusing on the use of antiplatelet agents and anticoagulants.

A variety of clinical and radiological variables were analyzed, including the Alberta Stroke Program Early CT Score (ASPECTS), the site of vascular occlusion (e.g., internal carotid artery, anterior cerebral artery, M1 or M2 segment of the middle cerebral artery, posterior cerebral artery, or basilar artery), and procedural factors. Procedural factors comprised the time from groin puncture to the completion of the intervention, the number of maneuvers performed, deployment of intra- or extracranial stents, intraprocedural drug administration, and the volume of contrast injected.

Following the procedure, reperfusion success was assessed using the modified Thrombolysis in Cerebral Infarction (mTICI) score. Blood pressure (BP) was consistently monitored throughout the intervention, with systolic and diastolic BP values extracted from the anesthesia operative reports and analyzed for their mean values.

Data distribution was tested using the Shapiro-Wilk test. Categorical variables were analyzed with chi-squared tests, while continuous variables were assessed using either two-sided t-tests (for normally distributed data) or Wilcoxon-Mann-Whitney U tests (for non-normally distributed data). Descriptive statistics are presented as frequencies and percentages for categorical variables and as medians with interquartile ranges (IQR) for continuous variables.

Identified variables with a p value of < 0.05 were included in a binary logistic regression analysis to examine their role in the occurrence of CIE. Additionally, the associations between clinical outcomes (mRS at discharge and mortality) and CIE were assessed using multivariable logistic regression, adjusted for key covariates such as age, sex, initial NIHSS score, rt-PA administration, and successful recanalization.

All calculations were performed using SPSS software (Version 24; SPSS Inc, Chicago, IL, USA). *P* values < 0.05 were regarded as statistically significant.

## Results

**Fig. 1:**
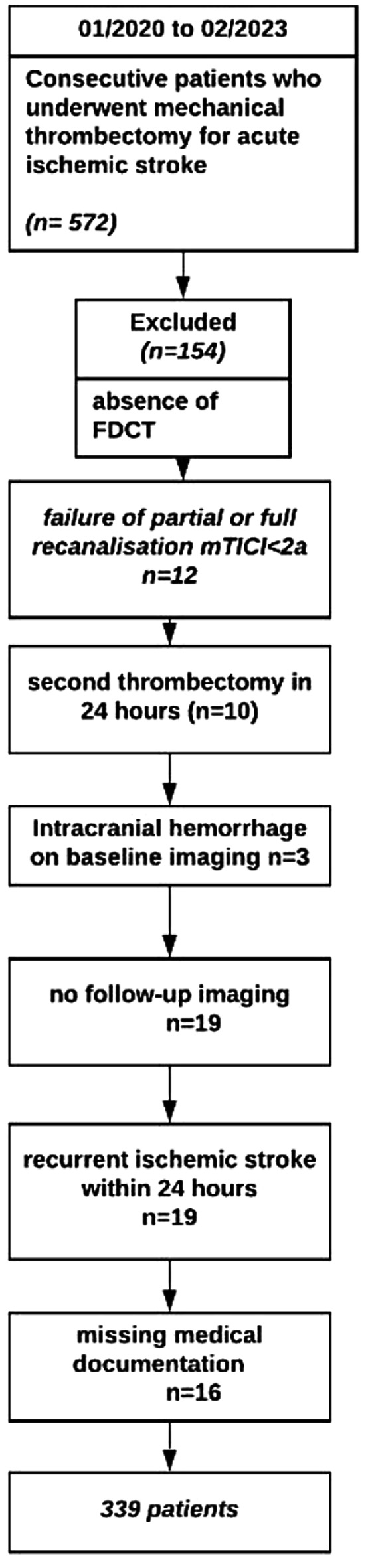
Patient flowchart with the inclusion and exclusion criteria

Five-hundred and seventy-two patients from the stroke registry who underwent EVT between January 2020 and February 2023 were analyzed. After applying the inclusion criteria, we ended up with 339 patients in total. The most common reasons for exclusion were the absence of FDCT after MT (*n* = 154), no follow-up imaging (*n* = 19), missing medical documentation (*n* = 16), recurrent ischemic stroke within 24 h (*n* = 19). The patient flowchart with inclusion and exclusion criteria is depicted in (Fig. 1).

Of the 339 patients, 16 patients developed CIE (4.7%) according to the implemented radiological and clinical criteria. Mean age of patients in both groups was similar (Patients without CIE: 79, patients with CIE: 81.4) with 49.8% and 56.3% of female patients, respectively. Baseline median NIHSS score was 14 (IQR: 9–18) and 12 (IQR: 11–18), with 42% and 28.6% of patients receiving IV tPA, respectively. Prestroke mRS was higher in the CIE group 1 (IQR: 0–1) vs. 0 (IQR:0–2), *p* = 0.033. Baseline clinical characteristics of patients with no CIE and patients with CIE are summarized in (Table 1).

**Table 1:** Baseline and clinical characteristics stratified by CIE

Group Median (lq-uq), [*N*], % (*n*/*N*)	Total 339	Patients without CIE 323	Patients with CIE 16	*P* Value
Age at stroke (years)				
	80 [69–87]	79 [68–87]	81.5 [75.2–86]	0.27
Sex				0.61
Male	49.9% (169)	50.2% (162)	43.8% (7)	
Female	50.1% (170)	49.8% (161)	56.3% (9)	
Baseline comorbidities Hypertension	73.7% (250)	74% (239)	68.8% (11)	0.64
Medical history of diabetes	27.4% (93)	26.6% (86)	43.8% (7)	0.13
Hyperlipidemia	66.1% (224)	65.3% (211)	81.3% (13)	0.18
Atrial fibrillation	41% (139)	42.2% (133)	37.5% (6)	0.77
Previous medication Antiplatelet therapy Anticoagulation therapy Initial ASPECTS NIHSS at admission	33% (112) 13.6% (46) 8[7–10] 14 [9–18]	33.7% (109) 13.9 (45) 8 [7–10] 14 [9.0–18.0]	18.8% (3) 6.3% (1) 9 [8–10] 12 [11–18.0]	0.213 0.381 0.399 **0.027**
Prestroke mRS	0 [0.0–2.0]	0 [0.0–2.0]	1 [0.0–1.0]	**0.033**
Intravenous thrombolysis	41.4% (137)	42% (133)	28.6% (4)	0.320
mRS at discharge	4 [2–5]	4 [2–5]	4 [2–5]	0.522
ASPECTS at discharge	7 [6–8]	7 [5–8]	7.5 [7–8]	**0.043**

NIHSS; National Institutes of Health Stroke Scale, ASPECTS; Alberta Stroke Program Early CT Score, mRS; modified Rankin scale. Values of *p* < 0.05 are considered statistically significant and are marked in bold

Glomerular filtration rate (GFR) was lower in the CIE group 60 mL/min/1.73m^2^ (IQR: 37–82) vs. 75 mL/min/1.73m^2^ (IQR:60–75), without statistical significance *p* = 0.127. Mean diastolic blood pressure was lower in the CIE group presenting greater variability 80 mm Hg (IQR:60–90) vs. 81 mm Hg (IQR:75–91), *p* = 0.020. Patients with CIE required significantly more device passes (maneuvers) to achieve successful recanalization 3(IQR:2–6) vs. 2(IQR:1–3), *p* = 0.033 and received significantly more contrast agent 200 mm (IQR:100–220) vs. 110 mm (IQR:90–150), *p* = 0.017. The duration of intervention was longer in the CIE group 145 min (IQR:78–193) vs. 85 min (IQR:57–125) without statistical significance *p* = 0.21. Extracranial stent angioplasty was necessary in 4 patients with CIE (rescue stenting). Additionally, two patients in the CIE group required intracranial stenting. One patient received a stent for stenosis in the distal M1 segment, while another underwent stenting for a long segmental dissection of the ICA, extending from C1 to C3. The latter case involved the placement of a proximal ICA stent combined with an overlapping distal flow-diverter. The analysis demonstrates statistically significant results for the deployment of intracranial stents in the CIE group (*p* = 0.047) and for the occlusion of the cervical ICA (*p* = 0.025). Intracranial hemorrhage was not increased in the CIE group.

Procedural and interventional characteristics of both patient groups are summarized in (Table 2).

**Table 2:** Procedural and interventional characteristics stratified by CIE

(Mean ± STD) [N], % (n/N) or median (IQR	Total 339	Patients without CIE 323	Patients with CIE 16	P value
GFR (mL/min/1.73 m^2^)	75[57–80]	75[60–75]	60[37–82]	0.127
Glucose (mg/dL)	123[104–142]	122.5[104–143]	126[111–131]	0.135
Mean systolic blood pressure (mm Hg) Mean diastolic blood pressure (mm Hg) Mean heart rate (BPM)	150[130–167] 80[75–91] 81[70–93]	150[130–167] 81[75–91] 82[71–94]	150[140–160] 80[60–90] 70[60–90]	0.065 **0.020** 0.099
Intracranial stent Extracranial stent Maneuver count Injected Contrast volume (mm) Duration of intervention	3.6% (12) 13.9% (43) 2[1–4] 110 (90–160) 86[57–125]	3.1% (10) 13.4% (43) 2[1–3] 110[90–150] 85[57–125]	12.5% (2) 25% (4) 3[2–6] 200[100–220] 145[78–193]	**0.047** 0.19 **0.033** **0.017** 0.21
Tandem occlusion Cervical ICA Petrous/cavernous segment of ICA Terminal segment of ICA M1 M2 ACA PCA BA	12% (40) 9.1% (31) 3.6% (12) 12.8% (43) 50.4% (171) 35% (118) 3% (10) 0% (0) 2.9% (10)	11.6% (37) 8.4% (27) 3.7% (12) 13.1(42%) 51.4% (166) 34.3% (110) 2.8% (9) 0% (0) 3.7% (10)	18.8% (3) 25% (4) 0% (0) 6.3% (1) 31.3% (5) 50% (8) 6.3% (1) 0% (0) 0% (0)	0.39 **0.025** 0.43 0.42 0.116 0.198 0.428 0.67
mTICI 2a		3.1% (10)	6.3% (1)	**0.018**
2b 2c 3		35% (113) 11.1% (36) 50.8% (164)	6.3% (1) 31.3% (5) 56.3% (9)	
Hemorrhagic transformation/Parenchymal hematoma	13.8% (47)	13.9% (45)	12.5% (2)	0.84

NIHSS; National Institute of Health Stroke score, ASPECTS; Alberta Stroke Program Early CT Score, IV- t-PA; intravenous tissue plasminogen activator, ICA; internal carotid artery, ACA; anterior cerebral artery, PCA; posterior cerebral artery, mTICIs; modified treatment in cerebral infarction score. Values of *p* < 0.05 are considered statistically significant and are marked in bold

**Fig. 2:**
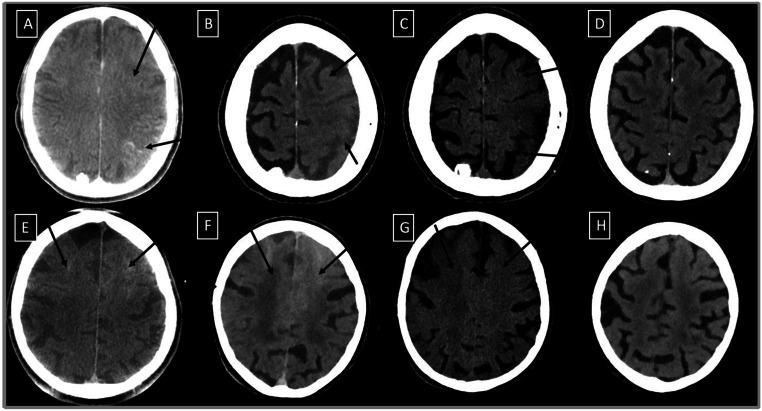
Immediate postinterventional FDCT scans and follow-up DECTs of two patients demonstrating reversible changes seen in contrast-induced encephalopathy: (**A**), (**B**) cerebral edema and sulci effacement and localized subarachnoid hyperdensities in the left frontoparietal region on FDCT and first follow-up DECT, respectively, these changes are confirmed with the virtual non-contrast scan (VNC) (**C**) and are fully reversible on second follow-up DECT (**D**) after 72 h. (**E**), (**F**) cerebral sulci effacement and cortical enhancement of the right frontal region extending contralaterally on FDCT and first follow-up DECT, respectively, these changes are confirmed with the virtual non-contrast scan (VNC) (**G**) and are fully reversible on second follow-up DECT (**H**) after 72 h

In addition to the aforementioned radiological criteria for the diagnosis, we observed three cases, in which the reversible brain edema extended to the contralateral hemisphere in the parafalcine region. The underlying occlusions of these cases were the M1 segment in two patients and the cervical ICA in one patient. (Fig. 2) presents examples of changes accompanying CIE recognized on FDCT and verified on follow-up DECTs.

**Fig. 3:**
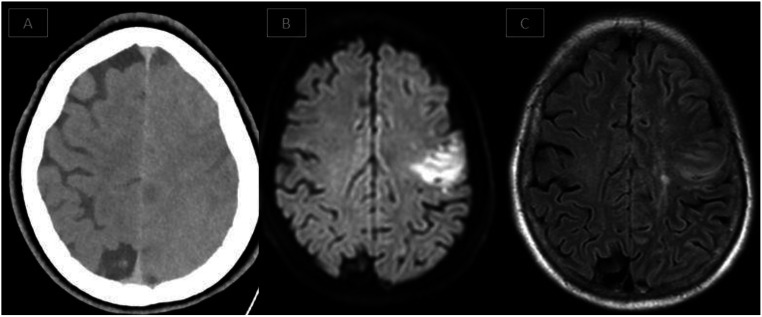
Immediate postinterventional FDCT scan and follow-up MRI of patient demonstrating reversible changes seen in contrast-induced encephalopathy: (**A**) cerebral edema and sulci effacement of the left hemisphere as seen on FDCT. (**B**) and (**C**) DWI and FLAIR sequences demonstrating diffusion restriction within the infarct core, along with diffuse cortical hyperintensity on FLAIR imaging extending beyond the infarcted territory, without corresponding diffusion restriction. This was accompanied by left hemispheric swelling

In this study, 4 out of 16 patients diagnosed with CIE underwent MRI.

(Fig. 3) presents examples of changes accompanying CIE recognized on FDCT and MRI.

Two of the patients from the CIE group required intubation due to their soporific status and respiratory instability. They died later due to multi-organ failure and had originally underlying high cardiovascular risk profile.

The individual peri- and postinterventional characteristics of the CIE patients are listed in (Table 3).

**Table 3:** Demonstrates individual peri- and postinterventional characteristics of each of the CIE patients

	Intervention time	Occlusions site	Maneuver count	Contrast agent volume	Extracranial stent	Intracranial stent	mTICI	mRS at discharge	GFR	Mean diastolic blood pressure	Mean systolic blood pressure	Clinical worsening	
1 2 3 4	120 105 115 185	M1-M2 M2 M1 segment ACA- PCA- ICA cervical-M1 segment	4 3 3 10	200 85 160 220	No No No Yes	Yes No No No	3 2c 3 3	2 3 3 5	82 90 31 90	80 60 130 130	130 150 170 180	GCS 10- seizure GCS 10 GCS 8 soporific Worsening of hemiparesis- GCS 10	
5 6 7 8 9 10	108 47 41 69 60 218	M1- ICA terminus ICA terminus M2 M1 Cervical ICA Cervical ICA	10 5 1 5 1 3	220 200 100 230 60 200	No No No No Yes Yes	No No No No No No	3 2c 3 2c 3 2c	1 4 1 4 6 3	82 83 49 31 37 90	120 90 60 60 70 80	150 160 120 140 120 150	GCS 7 Worsening of hemiparesis GCS 8 soporific GCS 8 soporific GCS 8 soporific- seizure GCS 7	
11 12 13 14 15 16	335 161 195 170 160 130	ICA dissection M2 M2 M2 M2 M2	5 6 7 3 2 5	370 250 220 110 75 125	Yes No No No No No	Yes No No No No No	3 2a 3 2c 3 2b	5 4 3 3 6 4	60 55 70 28 39 63	60 80 120 90 70 80	180 140 170 150 140 160	GCS 10- seizure Worsening of hemiparesis GCS 8 soporific Worsening of hemiparesis- seizure GCS 7 Worsening of Aphasia- seizure	

ICA; internal carotid artery, ACA; anterior cerebral artery, PCA; posterior cerebral artery, mTICIs; modified treatment in cerebral infarction score. Values of *p* < 0.05 are considered statistically significant and are marked in bold

*Mean systolic and diastolic blood pressure (mm Hg).*

GFR; Glomerular filtration rate mL/min/1.73m^2^

**Table 4:** Predictors of contrast-induced encephalopathy

	Odds ratio	95% CI	*P*-value
Discharge ASPECTS	1.25	0.88 to 1.76	0.208
Baseline NIHSS	0.97	0.86 to 1.1	0.656
Number of device passes	1.51	1.13 to 2.01	**0.005**
Cervical ICA occlusion	2.84	0.27 to 29.8	0.384
Injected contrast volume	1.00	0.99 to 1.01	0.341
Intracranial Stent	1.46	0.11 to 18.60	0.765
Mean diastolic blood pressure	1.00	1.00 to 0.96	0.914

Values of *p* < 0.05 are considered statistically significant and are marked in bold

Ordinal logistic regression analysis demonstrated increased odds of developing CIE in patients with high number of device passes (OR = 1.51; 95% CI, 1.13–2.01). The model failed to estimate the effect of other possible predictors such as occlusion of cervical ICA or intracranial stent with (OR = 2.84; 95% CI, 0.27–29.8) and (OR = 1.46; 95% CI, 0.11–18.60) respectively. This could be due to the rarity of the investigated event CIE (16/339). Results of regression analysis are summarized in (Table 4).

After adjusting for relevant covariates, multivariable logistic regression didn’t find meaningful association between patients with CIE and unfavorable clinical outcomes (mRS > 3) (OR: 1.04; 95% CI: 0.34–3.21; *P* = 0.9) or mortality (OR: 1.12; 95% CI: 0.23–5.41; *P* = 0.89). As indicated previously, wide confidence interval indicates a high level of uncertainty.

## Discussion

In this study, we report an incidence rate of CIE of 4.9% (16 out of 339) patients after MT. This rate is higher than that reported in a previous study, which identified 7 cases among 421 patients (1.7%) undergoing MT across two stroke centers. In that study, follow-up imaging was performed based on clinical judgment, with the earliest occurring 12 h post-intervention. To the best of our knowledge, this is the first investigation to include such a large number of patients from a single stroke center and to utilize FDCT for detecting CIE directly in the angio-suite. This more systematic approach likely increased sensitivity for detecting cases that may have otherwise gone unrecognized.

CIE is an elusive entity to diagnose in patients with AIS, as it requires distinguishing it from other post-thrombectomy complications such hemorrhagic transformation and recurrent ischemic stroke. In this investigation symptoms most commonly appeared either immediately or within a few hours after MT, with some showing gradual progression over time. However, the radiological changes were evident directly on FDCT in the angio-suite. This discrepancy may partly be attributed to the fact that all MT procedures at this stroke center were performed under general anesthesia, which could mask the recognition of clinical deterioration during anesthesia withdrawal. The range of neurological deficits varied, depending on the arterial territory involved [6, 8]. CIE resolved with supportive management including IV hydration and administration of steroids. Anticonvulsive treatment was administered to patients who developed seizures. While the discharge mRS remained unchanged, a limited number of patients in our study who experienced CIE achieved unfavorable outcomes. This is more likely attributable to their extended stay in intensive care and pre-existing cardiovascular risk factors.

Although GFR was lower in the CIE group, this difference did not reach statistical significance (*p* = 0.127). Nonetheless, this suggests that impaired renal function may play a role in the reduced clearance of contrast medium, which can increase its osmolality and neurotoxic effects [5].

Patients with CIE required significantly more device passes *p* = 0.033, and received a significantly higher volume of contrast agent *p* = 0.017. Both factors reflect a greater procedural complexity and therefore increased exposure to contrast medium. While the intervention duration was longer in the CIE group, this difference was not statistically significant (*p* = 0.21). Additionally, deployment of intracranial stents *p* = 0.047 and occlusion of the cervical ICA (0.025) were apparent in the CIE group. The implemented regression analysis demonstrated increased odds of developing CIE in patients with high number of device passes (OR = 1.51, *p* = 0.005). Nevertheless, regression analysis failed to identify the effect of further factors such as amount of administered contrast agent in development of CIE, this could be attributed to the low incidence of the investigated event. It is therefore possible that, in our cohort, device passes may serve as a proxy for increased contrast agent exposure and other cumulative procedural stressors. This is in line with the investigation by Chu et al. in which the amount of administered contrast agent was comparable between patients with and without CIE and regression analysis couldn’t also identify it as a predictor of CIE [6].

In the context of AIS, the integrity of BBB is compromised, resulting in increased permeability [13]. Additionally, intra-arterial contrast has been shown to induce endothelial cell contraction, leading to the opening of tight junctions [14]. Furthermore, greater procedural complexity often necessitates a higher number of device passes to achieve recanalization. Consequently, the navigation and manipulation of microcatheters and stent retrievers during MT can amplify shear stress, further disrupting the integrity of the BBB [15]. This disruption leads to contrast leakage, direct toxicity, and subsequent cerebral edema. However, it remains unclear why only some patients develop CIE while others do not, even when procedural complexity is similar.

Lower mean diastolic blood pressure was observed in the CIE group, *p* = 0.020, which could reflect compromised cerebral autoregulation. Diastolic pressure reflects perfusion during cardiac relaxation, which is critical for sustaining microvascular flow [16]. Inadequate perfusion in the context of procedural hypotension or poor collateral status—can exacerbate BBB disruption, thus increasing the risk of contrast leakage and cerebral edema.

In this investigation, in addition to previously reported changes radiologically, we observed three cases, in which the reversible brain edema extended to the contralateral hemisphere in the parafalcine region. These cases occurred in patients with underlying MCA and ICA occlusions. This gives the impression of changes which may be seen in cerebral hyperperfusion syndrome (CHS). There are many case reports in the literature, in which CIE or CHS were used interchangeably to describe radiological changes accompanying clinical worsening after MT [17, 18], percutaneous transluminal angioplasty of the MCA [19] or stenting of the ICA [20]. In these aforementioned patients, repeated attempts at access and reperfusion may have allowed contrast to reflux or redistribute through the circle of Willis, including into the ACA and contralateral MCA territories.

This study has several limitations. First, while FDCT is routinely performed following mechanical thrombectomy (MT) for anterior circulation occlusions, its application in posterior circulation occlusions is limited due to inherent imaging challenges in the posterior fossa—particularly beam-hardening artifacts that reduce diagnostic utility. Second, the low incidence of CIE in our cohort limits the statistical power of the analysis and constrains the ability to explore additional covariates as potential predictors. Third, the absence of standardized diagnostic criteria for CIE remains a broader limitation in the field. To mitigate the risk of misclassification, we conducted a thorough retrospective review of all pre- and post-thrombectomy neuroimaging.

Although radiological evaluation was not conducted in a blinded fashion—owing to the retrospective and observational nature of this study—our imaging assessments closely reflect real-world clinical workflows. Future research should aim to incorporate blinded image reviews and establish reference standards in order to evaluate diagnostic performance metrics such as sensitivity and specificity for FDCT and DECT in detecting CIE. While MRI remains the gold standard for definitive infarct assessment, its routine use was limited in our cohort due to clinical constraints. Nonetheless, incorporating standardized MRI protocols in future studies would improve diagnostic accuracy of CIE.

## Conclusion

The diagnosis of CIE can be complicated by overlapping features with other post-EVT conditions, such as CHS, BBB disruption, or ischemic stroke progression. A thorough review of imaging findings and clinical context is crucial for accurate identification.

This investigation emphasizes that radiological changes associated with CIE are evident immediately on post-interventional FDCT. This finding carries significant clinical implications, including the need for more intensive post-interventional monitoring and perhaps prophylactic treatment for patients with high-profile cardiovascular risk factors, even before the onset of clinical deterioration.

## Data Availability

No datasets were generated or analysed during the current study.
